# Nutrition- and feeding practice-related risk factors for rapid weight gain during the first year of life: a population-based birth cohort study

**DOI:** 10.1186/s12887-020-02391-4

**Published:** 2020-11-05

**Authors:** Annelie Lindholm, Stefan Bergman, Bernt Alm, Ann Bremander, Jovanna Dahlgren, Josefine Roswall, Carin Staland-Nyman, Gerd Almquist-Tangen

**Affiliations:** 1 School of Health and Welfare, Halmstad University, Kristian IV:s väg 3, 301 18 Halmstad, Sweden; 2 Research and Development Center Spenshult, Halmstad, Sweden; 3 Primary Health Care Unit, Department of Public Health and Community Medicine, Institute of Medicine, The Sahlgrenska Academy, University of Gothenburg, Gothenburg, Sweden; 4 Department of Pediatrics, Institute of Clinical Sciences, The Sahlgrenska Academy, University of Gothenburg, Gothenburg, Sweden; 5 Department of Regional Health Research, University of Southern Denmark, Odense, Denmark; 6 Department of Pediatrics, Halland Hospital, Halmstad, Sweden; 7 Child Health Care Unit, Region Halland, Halmstad, Sweden

**Keywords:** Bottle-feeding, Breastfeeding, Growth, Infant, Milk cereal drink, Nutrition, Obesity, Overweight, Pediatrics, Rapid weight gain

## Abstract

**Background:**

Rapid weight gain (RWG) during infancy increases the risk of excess weight later in life. Nutrition- and feeding practices associated with RWG need to be further examined. The present study aimed to examine nutrition- and feeding practice-related risk factors for RWG during the first year of life.

**Methods:**

A population-based longitudinal birth cohort study of 1780 infants, classified as having RWG or non-RWG during 0–3-4, 0–6 and 6–12 months. RWG was defined as a change > 0.67 in weight standard deviation scores. Associations between nutrition- and feeding practice-related factors and RWG were examined with logistic regression models.

**Results:**

Of the participating infants, 47% had RWG during 0–3-4 months, 46% during 0–6 months and 8% during 6–12 months. In the fully adjusted models, bottle-feeding at birth and at 3–4 months and nighttime meals containing formula milk were positively associated with RWG during 0–3-4 months (*p* < 0.05 for all). Breastfeeding at 3–4 months and nighttime meals containing breast milk were negatively associated with RWG during this period (*p* < 0.001). Bottle-feeding at birth, 3–4 and 6 months and nighttime meals containing formula milk at 3–4 months were positively associated with RWG during 0–6 months (*p* < 0.01 for all). Breastfeeding at 3–4 and 6 months was negatively associated with RWG (*p* < 0.01). During 6–12 months, only bottle-feeding at 3–4 months was positively associated with RWG (*p* < 0.05).

**Conclusions:**

RWG was more common during the first 6 months of life and bottle-feeding and formula milk given at night were risk factors for RWG during this period.

**Supplementary information:**

**Supplementary information** accompanies this paper at 10.1186/s12887-020-02391-4.

## Background

The global prevalence of childhood overweight and obesity has grown dramatically in recent decades [1], and even the youngest age groups are affected [2]. The “first 1,000 days” of a child’s life, beginning at conception and ending on their second birthday, has been identified as an important period for the establishment of obesity and associated diseases affecting life-long health [3].

One identified risk factor for later overweight that has gained much attention in recent research is rapid weight gain (RWG), seen as upward centile crossing in weight growth charts and defined as a change > 0.67 in weight standard deviation scores (SDS) [4, 5].

RWG during the first two years of life has in a number of studies been associated with overweight or obesity in both childhood and adulthood [3–5]. One review revealed that 45 of 46 studies reported a positive association between higher infancy weight or RWG and later childhood overweight [3]. Several studies have attempted to determine during what time period in infancy or childhood RWG predicts later adiposity but have come to mixed results [6, 7] although, early infancy has been suggested as a critical period [5].

Observed risk factors for rapid weight gain operates both before and during pregnancy as well as early in life. These include maternal pre-pregnancy overweight [8–12] and excessive maternal gestational weight gain [9–11], maternal and paternal overweight [8], maternal smoking during pregnancy [8, 13], maternal smoking after birth or history of smoking [13], smoking in the same room as the child [8], male sex in the child [13, 14], younger gestational age, first born status [13] and low socioeconomic status [15]. Besides these risk factors, a number of nutrition- or feeding practice-related factors acting early in life have been associated with RWG [16–18]. Breastfed infants have a slower weight gain trajectory compared to infants fed on formula milk, at least in Westernized settings [17]. The milk type (i.e., formula milk versus breast milk), and mode of feeding (i.e., directly from the breast versus from a bottle), both independently and in combination with each other may be of importance regarding associations between early feeding and RWG [18, 19]. Infants fed on formula milk have been shown to have higher intakes of protein and energy and a faster weight gain than infants fed on breast milk [19], and formula milk with a high protein content promotes RWG [20]. In terms of mode of feeding, bottle-feeding has been associated with RWG regardless of whether the bottle contained formula milk or expressed breast milk [18].

In the Swedish context, milk cereal drink (MCD) (“gruel”) which is a liquid-based complement to breast milk recommended from the age of 6 months [21–23], is also a factor that may have an impact on RWG. MCD is commercially available as a ready mix [24]. It is made from grains and dehydrated skimmed milk, mixed with hot water. The most common grains in gruel are oats but versions with wheat, rye, semolina and corn are also available. It contains approximately 65 kcals/100 mL, 2.7 g protein, 8.3 g carbohydrates and 2.1 g fat [23]. Positive associations between MCD consumption and overweight have been found [21, 22, 24].

The early establishment of excess weight demands interventions already in infants and toddlers [25]. Changing modifiable risk factors early in life may be an effective way of reducing the prevalence of overweight in children [25, 26]. Early nutrition and feeding practices have been identified as modifiable risk factors for overweight and obesity [25], but in order to develop early interventions, more research is needed regarding current dietary practices and RWG in infants [4, 27].

The aim of the present study was to examine nutrition- and feeding practice-related risk factors for RWG during the first 0–3-4 months, 0–6 months and 6–12 months of the first year, adjusted for biological (birth weight and sex), socioeconomic and parental health-related risk factors for RWG or childhood overweight or obesity.

## Methods

### Study population and design

This longitudinal birth cohort study is a part of the ongoing population-based birth cohort study, the Halland Health and Growth Study (H^2^GS). The H^2^GS included at baseline 2666 infants, 1349 boys and 1317 girls, born in the County of Halland, in south-western Sweden, between 1 October 2007 and 31 December 2008. During the period the data was collected, there were 3860 births in the county, all infants were eligible to take part in the study, without any exclusions and 69% agreed to participate. The families were recruited at their first visit to the child health care centers (CHCC), which in Halland serves about 98.4% of the infants living in the county. Measurements of length, weight and waist circumference were carried out by trained child health care nurses at 0–1, 3–4, 6 and 12 months. In connection with the measurements, the parents filled in questionnaires regarding their infant’s food, lifestyle and background data. The study protocol, the recruitment process and the representativeness of the sample have been reported in detail elsewhere [28].

**Fig. 1:**
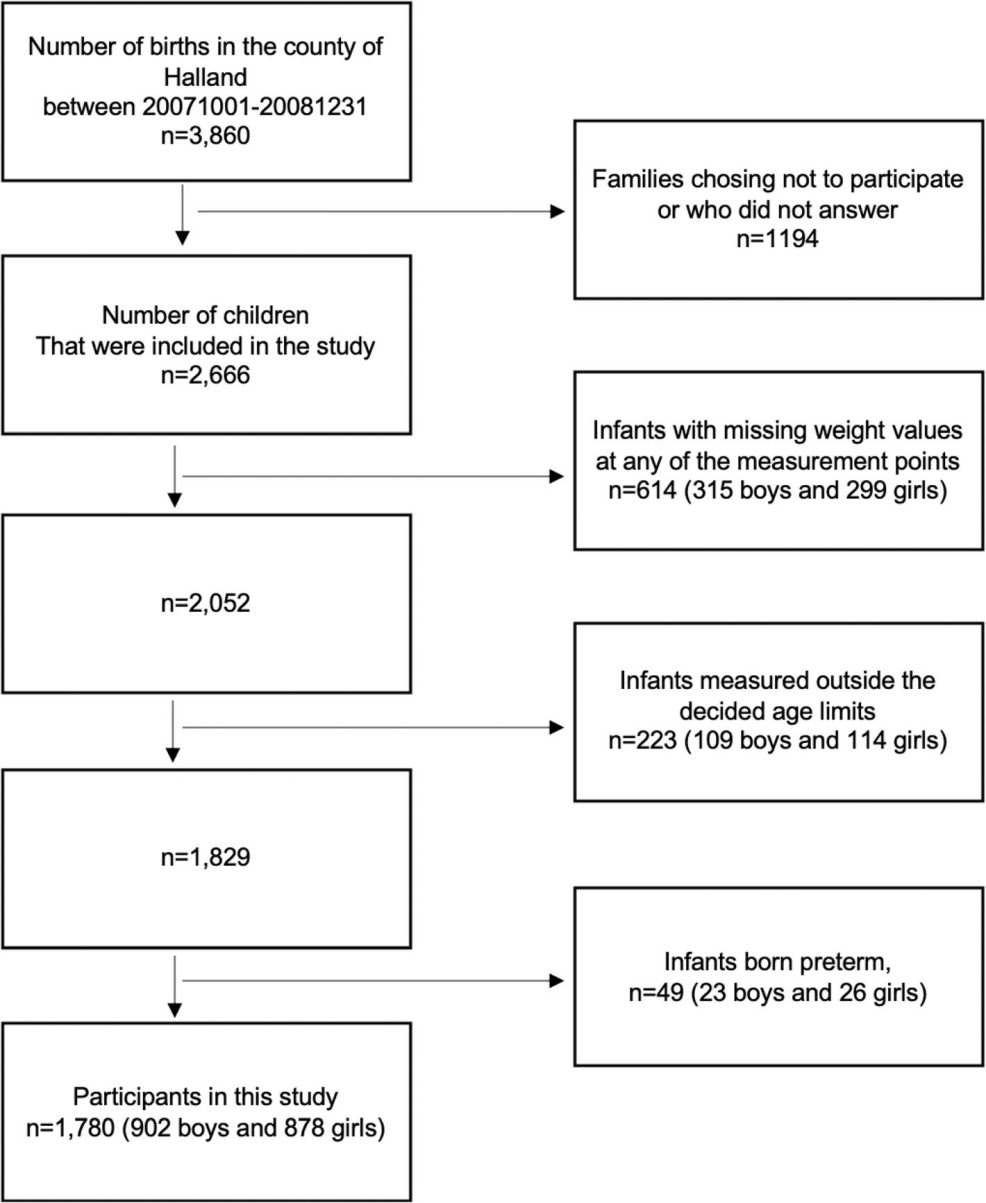
Flowchart over the population included in this study

In this part of the study with a focus on RWG during 0–3-4, 0–6 and 6–12 months, only infants with weight values at 0, 3–4, 6 and 12 months and information regarding gestational age (GA) were included, excluding 315 boys and 299 girls. Infants measured outside the decided age limits, 0–31 days for the measurement point 0–1 month, ±1.5 month at 3–4 and 6 months and ± 2.5 months at 12 months were also excluded, 109 boys and 114 girls. Finally, infants born preterm, 23 boys and 26 girls were excluded. The final study population consisted of 1780 infants, 902 boys and 878 girls (Fig. 1). The excluded 886 infants had a significantly lower birth weight, 3420 g versus 3576 g (*p* < 0.001) but the balance between boys and girls was the same as among the infants included in the study.

### Definition of rapid weight gain

Birth weight, birth length and GA were collected from medical records where birth weight was reported to the nearest gram and birth length to the nearest half centimeter. Weight measurements were performed on calibrated scales with one measurement per infant. GA at birth was based on the date of the last menstrual period confirmed by antenatal ultrasound reports. Measurements of weight were conducted when the infants were 0–1, 3–4, 6, and 12 months old. Infants were weighed in a supine position on baby scales and without clothes. Crude weight values were transformed to sex- and age-specific standard deviation scores (SDS), using the estimated mean and standard deviation (SD) functions based on Swedish reference data [29]. RWG was defined as a change > 0.67 in weight SDS. This change in SDS in standard growth curves represents the distance between two adjacent centile lines. According to size at birth, the infants were considered as large for gestational age (LGA), appropriate for gestational age (AGA), or small for gestational age (SGA). LGA was defined as birth weight or birth length ≥ 2 SDS for GA, and SGA was defined as birth weight or birth length ≤ − 2 SDS for GA, according to Swedish reference standards [30].

### Nutrition and feeding practices and potential risk factors for RWG

Bottle-feeding was reported in the questionnaires at every measurement point and the parents were asked if their child used a feeding-bottle or not. Breastfeeding was reported at every measurement point and divided into predominant, partial or no, breastfeeding, based on answers from the questionnaires where the parents at every time point were asked if the child was breastfed or not. The parents who answered yes, were asked how many times per day, with three answering alternatives: 1–5 times/day, 6–10 times/ day or more than 10 times/day. Infants who were breastfed 6–10 times /day or more were considered predominantly breastfed, while the ones breastfed 1–5 times/ day were considered partly breastfed. Infants below 6 months of age, where the parents answered no to breastfeeding were considered formula fed. MCD consumption was reported at 6 and 12 months. Nighttime meals and their content, breastmilk, formula milk, MCD (from 6 months) or water were reported at 3–4 and 6 months. Background data regarding parental weight, height, maternal gestational weight gain, parental education (with university as the highest degree), parental smoking (current), diabetes mellitus and cardiovascular disease were recorded at the first visit to the CHCC in a questionnaire answered by the parents. Regarding the diseases, the parents were asked in the questionnaire if they had been treated for diabetes mellitus or cardiovascular disease. In the Swedish context cardiovascular disease includes hypertension, ischaemic heart disease and stroke.

Based on previous research regarding RWG or overweight/obesity [3, 8, 13, 15, 18, 31], and available data in this infant cohort, four potential nutrition- or feeding practice-related risk factors were examined: breastfeeding (where no breastfeeding was considered as a risk), MCD consumption, bottle-feeding and nighttime meals. These four risk factors were adjusted for the following factors: birth weight, sex, maternal weight before pregnancy, maternal gestational weight gain, paternal weight at the first measurement point, maternal and paternal smoking, maternal and paternal education and maternal and paternal diabetes mellitus and cardiovascular disease. Maternal BMI before pregnancy and paternal BMI at the first measurement point were also examined as risk factors for RWG. Since only paternal BMI but both maternal and paternal weight showed significant association with RWG, and maternal weight gain also was included as another potential risk factor, the weight values were used in the predictive models. Male sex, maternal smoking, maternal overweight before pregnancy, excess maternal gestational weight gain, paternal overweight and low or high birth weight in the infant have all been associated with RWG and overweight or obesity in previous research [3, 8, 9, 13]. Paternal smoking and parental education were examined as indicators of socioeconomic level since low socioeconomic level has been associated with RWG [3, 15]. Diabetes mellitus and cardiovascular disease were examined since they often are consequences of overweight or obesity. These 13 risk factors were grouped in three models: biological, socioeconomic or parental health related. Model 1 consisted of the child related factors, birth weight and sex, model 2 consisted of factors related to the parents socioeconomic situation, parental education and parental smoking and model 3 consisted of factors related to the health of the parents, maternal weight before pregnancy, maternal gestational weight gain, paternal weight and parental diabetes mellitus and cardiovascular disease.

### Statistics

The percentage of children with RWG during the 0–3-4 month-, 0–6 month- and, 6–12-month periods and children that had RWG during all of these time periods were analysed. The percentage of boys and girls and children born LGA or SGA in each group and if they had RWG or not during the three time periods were analysed. In each group, mean weight gain with standard deviations were calculated. Regarding nutritional factors, the percentage of children predominantly breastfed, bottle fed and exclusively formula-fed (at 0 and 3–4 months) during the different measurement points and the percentage of children given MCD at the time points 6 and 12 months were analysed. Data was not imputed, and missing values were treated as missing. Logistic regression analyses were performed and first the biological, socioeconomic and parental health-related risk factors aimed to examine in relation to nutrition and feeding- practices were examined one by one in a model adjusted for birth weight and sex. The dependent variable in all those analyses was RWG versus non-RWG (nRWG) during 0–3-4 months, 0–6 months and 6–12 months respectively. Independent variables were birth weight, sex, maternal education, paternal education, maternal smoking, paternal smoking, maternal weight before pregnancy, maternal BMI before pregnancy, maternal gestational weight gain, paternal weight at the first measurement point, paternal BMI at the first measurement point and maternal and paternal diabetes mellitus and cardiovascular disease.

In logistic regression analyses examining associations between the nutrition- or feeding practice-related risk factors and RWG, three models were used where biological, socioeconomic and parental health-related risk factors were successively introduced. The dependent variable in all those analyses was RWG versus nRWG during 0–3-4 months, 0–6 months and 6–12 months respectively. Independent variables were breastfeeding at 0–1, 3–4 and 6 months, MCD consumption at 6 months (this variable was only used for examining associations with RWG during 6–12 months), bottle-feeding at 0, 3–4 and 6 months and nighttime meals at 3–4 and 6 months (the content of the nighttime meals was studied separately but with the same three models). Model 1 was adjusted for birth weight and sex, model 2 was additionally adjusted for parental smoking and parental education, with upper secondary school as reference. Model 3 was additionally adjusted for maternal weight before pregnancy, maternal gestational weight gain, paternal weight at the first measurement point and parental diabetes mellitus and cardiovascular disease. Each time period was examined separately in all logistic regression analyses. Sensitivity analyses including only the boys were made for each potential risk factor and for each model (reported in Additional file 1).

SPSS (IBM corp, Armonk, New York, v.25.0) was used for all statistical analyses. A *p* value < 0.05 was considered to be statistically significant. Conversions of crude weight values to SDS were made in Matlab (The MathWorks, Natick, Massachusetts, v.9.0.0.341360R2016a).

## Results

### Study population

Of the 1780 infants, 47% had RWG during 0–3-4 months, 46% during 0–6 months, and 8% during 6–12 months (Table 1). The percent of infants experiencing RWG during all three time periods was 2%. The infants with RWG during the first 3–4 months had a mean weight gain of 3.0 ± 0.6 kg compared to 2.2 ± 0.5 kg for infants with nRWG (*p* < 0.001). In the RWG-group 58% were boys and 42% were girls. The corresponding mean increase in weight during the 0–6 months period was 4.8 ± 0.7 kg for infants with RWG and 3.7 ± 0.5 kg for infants with nRWG (*p* < 0.001). Among the children experiencing RWG during this period, 53% were boys and 47% were girls. During the 6–12-month period, the corresponding mean increases in weight were 3.3 ± 0.5 kg and 2.0 ± 0.5 kg for infants with RWG and nRWG respectively (*p* < 0.001), and the RWG-group consisted of 50% boys and 50% girls. Of the infants with RWG during 0–3-4 months, 4.4% were born SGA, and 2.1% LGA. The corresponding values in the 0–6 months group were 4.4 and 1.8% and in the 6–12-month group 3.7 and 3% for SGA and LGA respectively. Among the infants born SGA, 69% had RWG during 0–3-4 months, 67% during 0–6 months and 9% during 6–12 months. In the infants born LGA, 22% had RWG during 0–3-4 months, 18% during 0–6 months and 5% during 6–12 months. At birth, 87% of the infants were predominantly breastfed. The corresponding values for the other time points were as follows, 67% at 3–4 months, 29% at 6 months, and 4.5% at 12 months. During the different time points, the following numbers of infants were bottle-fed: at birth, 24%, at 3–4 months, 46% at 6 months, 76% and at 12 months, 89%. At birth, 6% of the infants were exclusively formula-fed and at 3–4 months the corresponding number was 20%. At 6 months of age, MCD was given to 44% of the infants and at 12 months the corresponding number was 59%.

**Table 1:** Characteristics of the study population divided by rapid weight gain or non-rapid weight gain during 0–3-4, 0–6 and 6–12 months

***n*** (total) = 1780	RWG 0–3-4 months (*n* = 841)	nRWG 0–3-4 months (*n*=939)	RWG 0–6 months (*n* = 827)	nRWG 0–6 months (*n* = 953)	RWG 6–12 months (*n* = 136)	nRWG 6–12 months (*n* = 1644)
**Sex**						
Boys	484	418	437	465	68	834
Girls	357	521	390	488	68	810
**Birth weight**^**a**^						
MBW ± SD (g)	3443 ± 456	3696 ± 478***	3439 ± 449	3696 ± 482***	3535 ± 464	3586 ± 486^ns^
Missing (*n*)	23	24	21	26	4	43
**Size for gestational age**						
SGA (*n*)	37	17	36	18	5	49
AGA (*n*)	785	856	776	865	126	1515
LGA (*n*)	18	65	15	68	4	79
Missing (*n*)	1	1	0	2	1	1
**Gestational age**						
37^0^–37^6^ (*n*)	41	34	42	33	10	65
38^0^–40^6^ (*n*)	604	691	576	719	90	1205
41^0^–43^5^ (*n*)	196	214	209	201	36	374
Missing (*n*)	0	0	0	0	0	0
**Maternal age**						
< 25 (*n*)	128	116	117	127	18	226
25–35 (*n*)	560	660	564	656	94	1126
≥ 35 (*n*)	140	155	131	164	22	273
Missing (*n*)	13	8	15	6	2	19
**Mean weight gain**^**a**^						
0–3 m (kg)	3.0 ± 0.6	2.2 ± 0.5***				
0–6 m (kg)			4.8 ± 0.7	3.7 ± 0.5***		
6–12 m (kg)					3.3 ± 0.5	2.0 ± 0.5***

^a^For mean values in birth weight and mean weight gain, the groups with RWG was compared with the groups with nRWG. ***p < 0.001, ns, non-significant

*RWG* rapid weight gain, *nRWG* non-rapid weight gain, *MBW* mean birth weight; *SD* standard deviation, *ns* non-significant; *SGA* small for gestational age;

*AGA* appropriate for gestational age, *LGA* large for gestational age

### Nutrition, feeding practices and RWG during 0–3-4 months

In logistic regression analyses of risk factors for RWG during 0–3-4 months and adjusted for birth weight and sex, significant positive associations were found for maternal weight before pregnancy, maternal gestational weight gain, paternal weight and paternal BMI at the first measurement point (Table 2). In a sensitivity analysis including only the boys, maternal gestational weight gain and paternal weight showed significant positive associations with RWG, paternal BMI was nearly significant (*p* = 0.055) (Additional file 1).

**Table 2:** Logistic regressions over risk factors for rapid weight gain during 0–3-4 months, 0–6 months and 6–12 months

Risk factors	RWG/nRWG				RWG/nRWG				RWG/nRWG		n (total) = 1780	
	0–3-4 m (n)	OR	95% CI	p	0–6 m (n)	OR	95% CI	p	6–12 m (n)	OR	95% CI	p
**Sex**												
Boy	484/418	1	Ref		437/465	1	Ref		68/134	1	Ref	
Girl	357/521	0.47*	0.39, 0.58	**<0.001**	390/488	0.71*	0.58, 0.86	**0.001**	68/810	0.98*	0.68, 1.40	0.913
**Birth weight** Per kg bw	n/a	0.27^∋^	0.21, 0.34	**<0.001**	n/a	0.29^∋^	0.23, 0.36	**<0.001**	n/a	0.82^∋^	0.57, 1.20	0.305
**Maternal education**												
Upper secondary school	325/370	1	Ref		332/363	1	Ref		59/636	1	Ref	
Elementary school	49/44	1.11	0.70, 1.76	0.669	44/49	0.86	0.55, 1.37	0.533	6/87	0.75	0.31, 1.78	0.509
University	424/474	0.97	0.78, 1.20	0.755	404/494	0.85	0.69, 1.05	0.125	65/833	0.80	0.55, 1.16	0.243
Other	32/44	0.69	0.41, 1.16	0.159	35/41	0.83	0.50, 1.38	0.472	4/72	0.60	0.21, 1.71	0.340
**Paternal education**												
Upper secondary school	418/491	1	Ref		407/502	1	Ref		73/836	1	Ref	
Elementary school	45/52	1.10	0.70, 1.72	0.693	45/52	1.17	0.75, 1.82	0.496	12/85	1.68	0.88, 3.23	0.119
University	281/299	1.12	0.90, 1.40	0.317	281/299	1.20	0.96, 1.50	0.104	35/545	0.68	0.44, 1.05	0.079
Other	32/38	0.93	0.55, 1.55	0.772	32/38	0.99	0.60, 1.65	0.975	9/61	1.65	0.79, 3.47	0.183
**Maternal smoking**												
No	780/884	1	Ref		762/902	1	Ref		126/1538	1	Ref	
Yes	49/45	0.93	0.59, 1.48	0.761	52/42	1.13	0.72, 1.78	0.596	8/86	1.20	0.56, 2.55	0.638
**Paternal smoking**												
No	675/801	1	Ref		665/811	1	Ref		109/1367	1	Ref	
Yes	96/77	1.30	0.92, 1.83	0.136	95/78	1.35	0.96, 1.90	0.082	19/154	1.69	1.00, 2.83	**0.049**
**Maternal weight bp** Per kg bw	n/a	1.01	1.00, 1.02	**0.035**	n/a	1.01	1.00, 1.02	**0.008**	n/a	1.01	1.00, 1.02	0.486
**Maternal gwg** Per kg gw	n/a	1.02	1.01, 1.04	**0.013**	n/a	1.02	1.00, 1.04	0.081	n/a	1.00	0.97, 1.04	0.970
**Paternal weight** Per kg bw	n/a	1.02	1.01, 1.03	**<0.001**	n/ a	1.02	1.02, 1.03	**<0.001**	n/a	1.01	1.00, 1.03	0.184
**Maternal BMI bp**	n/a	1.10	0.98, 1.03	0.481	n/a	1.01	0.99, 1.04	0.260	n/a	1.01	0.97, 1.05	0.718
**Paternal BMI**	n/a	1.04	1.01, 1.08	**0.024**	n/a	1.05	1.02, 1.09	**0.004**	n/a	1.04	0.98, 1.10	0.237
**Maternal diabetes mellitus**												
No	814/916	1	Ref		804/926	1	Ref		131/1599	1	Ref	
Yes	1/6	0.32	0.04, 2.74	0.297	1/6	0.32	0.04, 2.73	0.295	1/6	2.20	0.26, 18.62	0.468
**Paternal diabetes mellitus**												
No	761/874	1	Ref		753/882	1	Ref		126/1509	1	Ref	
Yes	9/8	1.40	0.47, 4.14	0.543	6/11	0.81	0.28, 2.39	0.704	0/17	0.00	0.00, -	1.00
**Maternal cardiovascular disease**												
No	810/914	1	Ref		800/924	1	Ref		132/1592	1	Ref	
Yes	8/9	0.92	0.33, 2.55	0.874	7/10	0.74	0.27, 2.04	0.556	1/16	0.75	0.1, 5.72	0.783
**Paternal cardiovascular disease**												
No	763/869	1	Ref		751/881	1	Ref		125/1507	1	Ref	
Yes	5/8	0.55	0.16, 1.89	0.343	5/8	0.87	0.27, 2.84	0.822	1/12	1.12	0.14, 8.78	0.913

All risk factors were adjusted for birth weight and sex, except sex that was adjusted for birth weight and birth weight that was adjusted for sex, *adjusted for birth weight, ^∋^adjusted for sex

*RWG* rapid weight gain, *nRWG* non-rapid weight gain, *m* months, *n* number of subjects, *OR* odds ratios, *95% CI* 95% confidence intervals, *p p* value, *n/a* not applicable, *Kg* kilogram, *Per kg bw* per kg body weight, *Per kg gw* per kg gained weight, *Maternal gwg* maternal gestational weight gain, *BMI* body mass index

When focusing on nutrition- and feeding practice-related risk factors, bottle-feeding at 0–1 and 3–4 months were positively associated with RWG during 0–3-4 months (Table 3). These associations were found in model 1, when adjusted for birth weight and sex, in model 2, when additionally adjusted for parental smoking and education and in model 3, when additionally adjusted for maternal weight before pregnancy, maternal gestational weight gain, paternal weight at the first measurement point and parental diabetes mellitus and cardiovascular disease. Breastfeeding at 3–4 months was negatively associated with RWG in all three models. Nighttime meals were not significantly associated with RWG, but when specifically studying the content of the meals at night, formula milk was positively associated, and breast milk was negatively associated with RWG during this period (Table 4). In the sensitivity analysis, the results were similar, except that bottle feeding at 3–4 months did not reach significant values (Additional file 1).

**Table 3:** Logistic regressions over nutrition- and feeding practice-related risk factors for rapid weight gain during 0**–**3-4 months

Risk factors 0–3-4 m	**Model 1**				**Model 2**				**Model 3**		***n*** (total) = 1780	
	***n*** in model	OR	95% CI	***p***	***n*** in model	OR	95% CI	***p***	***n*** in model	OR	95% CI	***p***
**Breastfeeding**												
0 months	1713				1577				1355			
No		1	Ref			1	Ref			1	Ref	
Yes		0.68	0.44, 1.05	0.080		0.68	0.43, 1.08	0.098		0.65	0.39, 1.07	0.090
3**–**4 months	1710				1568				1347			
No		1	Ref			1	Ref			1	Ref	
Yes		0.57	0.44, 0.73	**< 0.001**		0.56	0.42, 0.73	**< 0.001**		0.52	0.39, 0.70	**< 0.001**
**Bottle-feeding**												
0 months	1635				1508				1302			
No		1	Ref			1	Ref			1	Ref	
Yes		1.84	1.45, 2.34	**< 0.001**		1.96	1.52, 2.52	**< 0.001**		1.93	1.46, 2.54	**< 0.001**
3**–**4 months	1278				1172				1004			
No		1	Ref			1	Ref			1	Ref	
Yes		1.37	1.08, 1.74	**0.011**		1.40	1.08, 1.81	**0.010**		1.42	1.07, 1.88	**0.014**
**Nighttime meals**												
3**–**4 months	1713				1571				1349			
No		1	Ref			1	Ref			1	Ref	
Yes		0.83	0.65, 1.06	0.127		0.85	0.66, 1.10	0.217		0.86	0.65, 1.14	0.300

Model 1, adjusted for birth weight and sex; Model 2, additionally adjusted for maternal and paternal education and maternal and paternal smoking; Model 3,

additionally adjusted for maternal weight before pregnancy, maternal gestational weight gain, paternal weight at the first measurement point and

maternal or paternal diabetes mellitus and cardiovascular disease

**Table 4:** Logistic regressions over content of nighttime meals and rapid weight gain during 0–3-4, 0–6 and 6–12 months

Risk factors	**Model 1**				**Model 2**				**Model 3**		***n*** (total) = 1780	
	***n*** in model	OR	95% CI	***p***	***n*** in model	OR	95% CI	***p***	***n*** in model	OR	95% CI	***p***
**Time period 0–3-4 months**												
**Nighttime meals 3–4 months**												
Breast milk	1713				1571				1349			
No		1	Ref			1	Ref			1	Ref	
Yes		0.66	0.53, 0.81	**< 0.001**		0.66	0.52, 0.82	**< 0.001**		0.64	0.50, 0.82	**< 0.001**
Formula milk	1713				1571				1349			
No		1	Ref			1	Ref			1	Ref	
Yes		1.83	1.38, 2.42	**< 0.001**		1.86	1.38, 2.49	**< 0.001**		1.90	1.38, 2.61	**< 0.001**
Water	1713				1571				1349			
No		1	Ref			1	Ref			1	Ref	
Yes		1.22	0.16, 9.45	0.849		1.08	0.14, 8.18	0.941		1.18	0.16, 8.94	0.873
**Time period 0–6 months**												
**Nighttime meals 3–4 months**												
Breast milk	1713				1571				1349			
No		1	Ref			1	Ref			1	Ref	
Yes		0.68	0.55, 0.84	**< 0.001**		0.67	0.54, 0.84	**< 0.001**		0.66	0.51, 0.84	**0.001**
Formula milk	1713				1571				1349			
No		1	Ref			1	Ref			1	Ref	
Yes		1.68	1.28, 2.21	**< 0.001**		1.72	1.29, 2.29	**< 0.001**		1.83	1.33, 2.50	**< 0.001**
Water	1713				1571				1349			
No		1	Ref			1	Ref			1	Ref	
Yes		1.23	0.16, 9.39	0.841		1.14	0.16, 8.36	0.898		1.18	0.16, 8.70	0.872
**Nighttime meals 6 months**												
Breast milk	1711				1569				1350			
No		1	Ref			1	Ref			1	Ref	
Yes		0.67	0.55, 0.82	**< 0.001**		0.68	0.55, 0.84	**< 0.001**		0.66	0.52, 0.83	**< 0.001**
MCD	1711				1569				1350			
No		1	Ref			1	Ref			1	Ref	
Yes		1.33	1.00, 1.78	0.054		1.21	0.88, 1.66	0.232		1.28	0.91, 1.79	0.158
Water	1711				1569				1350			
No		1	Ref			1	Ref			1	Ref	
Yes		1.41	0.71, 2.83	0.327		1.32	0.62, 2.79	0.474		1.20	0.54, 2.66	0.663
**Time period 6–12 months**												
**Nighttime meals 3–4 months**												
Breast milk												
No	1713	1	Ref		1571	1	Ref		1349	1	Ref	
Yes		1.18	0.80, 1.74	0.403		1.18	0.78, 1.77	0.436		1.38	0.87, 2.20	0.170
Formula milk	1713				1571				1349			
No		1	Ref			1	Ref			1	Ref	
Yes		0.80	0.47, 1.36	0.412		0.81	0.47, 1.40	0.445		0.66	0.35, 1.25	0.207
Water	1713				1571				1349			
No		1	Ref			1	Ref			1	Ref	
Yes		4.18	0.43, 40.56	0.217		4.86	0.49, 48.29	0.177		5.10	0.51, 51.48	0.168
**Nighttime meals 6 months**												
Breast milk	1711				1569				1350			
No		1	Ref			1	Ref			1	Ref	
Yes		0.79	0.55, 1.14	0.208		0.81	0.55, 1.18	0.273		0.92	0.60, 1.40	0.693
MCD	1711				1569				1350			
No		1	Ref			1	Ref			1	Ref	
Yes		1.08	0.64, 1.82	0.766		1.09	0.63, 1.89	0.753		0.99	0.53, 1.85	0.976
Water	1711				1569				1350			
No		1	Ref			1	Ref			1	Ref	
Yes		1.58	0.55, 4.55	0.394		0.74	0.17, 3.20	0.687		0.39	0.05, 2.96	0.388

Model 1, adjusted for birth weight and sex; Model 2, additionally adjusted for maternal and paternal education and maternal and paternal smoking; Model 3,

additionally adjusted for maternal weight before pregnancy, maternal weight gain during pregnancy, paternal weight at the first measurement point and maternal

and paternal diabetes mellitus and cardiovascular disease

*MCD* milk cereal drink, *RWG* rapid weight gain, *nRWG* non-rapid weight gain, *m* months, *n* number of subjects, *OR* odds ratios, *95% CI* 95% confidence

interval, *p* p value

### Nutrition, feeding practices and RWG during 0–6 months

In logistic regression analyses of risk factors for RWG during 0–6 months and adjusted for birth weight and sex, significant positive associations were found for maternal weight before pregnancy, paternal weight and paternal BMI at the first measurement point (Table 2). In the sensitivity analysis including only the boys, significant positive associations were found for all three parental weight variables but not for parental BMI (Additional file 1).

When focusing on nutrition- and feeding practice-related risk factors, bottle-feeding at 0–1, 3–4 and 6 months was positively associated with RWG during 0–6 months (Table 5). These associations were found in model 1, when adjusted for birth weight and sex, in model 2, when additionally adjusted for parental smoking and education and in model 3, when additionally adjusted for maternal weight before pregnancy, maternal gestational weight gain, paternal weight at the first measurement point and parental diabetes mellitus and cardiovascular disease. Breastfeeding at 3–4 and 6 months was negatively associated with RWG in all three models. Nighttime meals at 3–4 months in model 1 as well as at 6 months in all three models were also negatively associated with RWG. When specifically studying the content of what the infants were given at night, it was shown that breast milk at 3–4 months and at 6 months was negatively associated with RWG, whereas formula milk at 3–4 months was positively associated with RWG (Table 4). MCD given as nighttime meal at 6 months did not reach significance as a risk factor for RWG but was nearly significant in model 1 (*p* = 0.054). The sensitivity analysis showed similar results except that the associations for nighttime meals did not reach significant values (Additional file 1).

**Table 5:** Logistic regressions over nutrition- and feeding practice-related risk factors for rapid weight gain during 0–6 months

Risk factors 0–6 m	**Model 1**				**Model 2**				**Model 3**		***n*** (total) = 1780	
	***n*** in model	OR	95% CI	***p***	***n*** in model	OR	95% CI	***p***	***n*** in model	OR	95% CI	***p***
**Breastfeeding**												
0 months	1713				1577				1355			
No		1	Ref			1	Ref			1	Ref	
Yes		0.89	0.58, 1.37	0.590		0.89	0.56, 1.41	0.623		0.80	0.49, 1.32	0.389
3**–**4 months	1710				1568				1347			
No		1	Ref			1	Ref			1	Ref	
Yes		0.64	0.50, 0.82	**< 0.001**		0.63	0.48, 0.82	**0.001**		0.59	0.44, 0.79	**< 0.001**
6 months	1648				1513				1305			
No		1	Ref			1	Ref			1	Ref	
Yes		0.64	0.52, 0.78	**< 0.001**		0.64	0.52, 0.80	**< 0.001**		0.64	0.50, 0.82	**< 0.001**
**Bottle-feeding**												
0 months	1635				1508				1302			
No		1	Ref			1	Ref			1	Ref	
Yes		2.06	1.62, 2.61	**< 0.001**		2.15	1.67, 2.76	**< 0.001**		2.26	1.71, 2.97	**< 0.001**
3**–**4 months	1278				1172				1004			
No		1	Ref			1	Ref			1	Ref	
Yes		1.73	1.36, 2.20	**< 0.001**		1.70	1.31, 2.19	**< 0.001**		1.69	1.27, 2.23	**< 0.001**
6 months	1704				1563				1346			
No		1	Ref			1	Ref			1	Ref	
Yes		1.65	1.29, 2.10	**< 0.001**		1.66	1.29, 2.14	**< 0.001**		1.59	1.20, 2.10	**0.001**
**Nighttime meals**												
3**–**4 months	1713				1571				1349			
No		1	Ref			1	Ref			1	Ref	
Yes		0.78	0.62, 1.00	**0.049**		0.79	0.61, 1.03	0.081		0.83	0.63, 1.10	0.200
6 months	1711				1569				1350			
No		1	Ref			1	Ref			1	Ref	
Yes		0.73	0.59, 0.90	**0.003**		0.73	0.59, 0.91	**0.004**		0.74	0.58, 0.93	**0.011**

Model 1, adjusted for birth weight and sex; Model 2, additionally adjusted for maternal and paternal education and maternal and paternal smoking; Model 3,

additionally adjusted for maternal weight before pregnancy, maternal gestational weight gain, paternal weight at the first measurement point and

maternal or paternal diabetes mellitus and cardiovascular disease

*MCD* milk cereal drink, *RWG* rapid weight gain, *nRWG* non-rapid weight gain, *m* months, *n* number of subjects, *OR* odds ratios, *95% CI* 95% confidence interval, *p* p value

### Nutrition, feeding practices and RWG during 6–12 months

During 6–12 months, a significant positive association was found between paternal smoking and RWG (Table 2). No significant associations were found in the sensitivity analysis including only the boys (Additional file 1). When focusing on nutrition- and feeding practice-related risk factors, only bottle-feeding at 3–4 months reached significant values as a risk factor for RWG in all three models (Table 6). In the sensitivity analysis the results were similar, except that nighttime meals at 6 months of age were significantly negatively associated with RWG and when studying the content of the meals at night, breast milk given at 6 months of age was negatively associated with RWG in model 1 and 2 (Additional file 1).

**Table 6:** Logistic regressions over nutrition-and feeding practice-related risk factors for rapid weight gain during 6**–**12 months

Risk factors 6–12 m	**Model 1**				**Model 2**				**Model** 3		***n*** (total) = 1780	
	***n*** in model	OR	95% CI	***p***	***n*** in model	OR	95% CI	***p***	***n*** in model	OR	95% CI	***p***
**Breastfeeding**												
0 months	1713				1577				1355			
No		1	Ref			1	Ref			1	Ref	
Yes		0.90	0.43, 1.91	0.790		0.91	0.42, 1.96	0.810		0.80	0.35, 1.83	0.602
3–4 months	1710				1568				1347			
No		1	Ref			1	Ref			1	Ref	
Yes		1.17	0.73, 1.87	0.516		1.18	0.72, 1.93	0.503		1.35	0.78, 2.36	0.289
6 months	1648				1513				1305			
No		1	Ref			1	Ref			1	Ref	
Yes		1.22	0.83, 1.78	0.316		1.30	0.87, 1.96	0.202		1.50	0.95, 2.37	0.081
**MCD**												
6 months	1622				1494				1283			
No		1	Ref			1	Ref			1	Ref	
Yes		1.16	0.79, 1.68	0.452		1.14	0.77, 1.70	0.516		1.00	0.64, 1.57	0.985
**Bottle-feeding**												
0 months	1635				1508				1302			
No		1	Ref			1	Ref			1	Ref	
Yes		0.90	0.59, 1.39	0.647		0.87	0.56, 1.37	0.553		0.80	0.48, 1.32	0.382
3**–**4 months	1278				1172				1004			
No		1	Ref			1	Ref			1	Ref	
Yes		2.05	1.24, 3.39	**0.005**		2.08	1.22, 3.55	**0.008**		1.89	1.05, 3.42	**0.035**
6 months	1704				1563				1346			
No		1	Ref			1	Ref			1	Ref	
Yes		1.57	0.97, 2.54	0.069		1.43	0.87, 2.34	0.159		1.29	0.75, 2.22	0.354
**Nighttime meals**												
3**–**4 months	1713				1571				1349			
No		1	Ref			1	Ref			1	Ref	
Yes		0.97	0.63, 1.51	0.895		0.96	0.61, 1.51	0.855		1.14	0.67, 1.92	0.636
**Nighttime meals**												
6 months	1711				1569				1350			
No		1	Ref			1	Ref			1	Ref	
Yes		0.78	0.54, 1.12	0.178		0.78	0.53, 1.15	0.206		0.88	0.57, 1.35	0.549

Model 1, adjusted for birth weight and sex; Model 2, additionally adjusted for maternal and paternal education and maternal and paternal smoking;

Model 3, additionally adjusted for maternal weight before pregnancy, maternal weight gain during pregnancy, paternal weight at the first measurement point and

maternal and paternal diabetes mellitus and cardiovascular disease

*MCD* milk cereal drink, *RWG* rapid weight gain, *nRWG* non-rapid weight gain, *m* months, *n* number of subjects, *OR* odds ratio, *95% CI* 95%

confidence interval, *p* p value

## Discussion

In this longitudinal birth cohort study, positive associations between nutrition- and feeding practice-related risk factors and RWG during the first year were found.

During the first 0–3-4 months, 47% of the infants experienced RWG and during 0–6 months the corresponding number was 46%. Not so many studies have examined weight gain during the first six months of life, but in one study with Japanese children, born between 1988 and 2000, 22.7% had RWG between 0 and 3–4 months [32]. In a Brazilian study with children born during 1993–1994 the percentage of children experiencing RWG between 0 and 6 months was 41,9% [33]. In a Swedish study with children born 1984–1985 and an Australian study with children born between 1981 and 1984 the percentages were 25,4% [34] and nearly 22% respectively [35]. One possible explanation for the lower numbers in the studies from the eighties is that not so many children had overweight or obesity back then. However, our results have to be compared with studies of children born in Sweden and other countries during the same time period to confirm if our results are representative for the population and the time period. During 6–12 months, only 8% of the infants in this study experienced RWG. Although the most important period for RWG regarding the prediction of later overweight is unclear, RWG during the first 6 months has been associated with overweight or obesity later in life in earlier studies [33–36]. The first 6 months of infancy has been shown to coincide with a gain in fat mass rather than in lean mass [37], and RWG during a period with fat mass accumulation may be one explanation for the association between RWG and later overweight.

With the relatively large number of breastfed infants in this child cohort and earlier published research showing slower weight gain trajectories in breastfed infants compared to infants fed on formula [17], the number of infants experiencing RWG during 0–3-4 and 0–6 months may seem unexpectedly high. However, at birth, 24% of the infants were bottle-fed, and at 3–4 months and 6 months the corresponding numbers were 46% and 76% respectively. Bottle-feeding at 0–1, 3–4 as well as at 6 months remained as a risk factor for RWG during the first 0–3-4 and 0–6 months even after adjustment for other risk factors. The same association was found regarding bottle- feeding at 3–4 months and RWG during 6–12 months. Bottle-feeding has been positively associated with RWG in other studies as well [18, 31], and this regardless of whether the bottle contained expressed breast milk or formula milk [18]. Bottle-feeding has been suggested to give more control to the caregiver and to facilitate a more pressuring, non-responsive feeding style [38, 39], and this may be one explanation for the associations found. It has been shown that when formula milk-feeding mothers used bottles without visual or weight cues of the volume left in the bottle, their responsiveness to their infant’s cues increased [39]. Breastfeeding has been suggested to support the development of responsive feeding, and the hypothesis behind this association is that the mother cannot measure how much milk the infant consumes, and therefore has to trust the infant’s hunger and satiety cues [40]. This may be one explanation to the negative association between breastfeeding and RWG that we found in our study. Responsive feeding has shown promising results regarding a reduced risk of RWG and adiposity in early childhood, although the best way to promote responsive feeding and the reasons behind suggested associations between breastfeeding and maternal responsiveness need to be further investigated [40, 41]. Other reasons for associations between bottle-feeding and RWG that have been suggested are a large bottle size [31, 42], feeding on a schedule [14], as well as infant initiated bottle emptying [31, 43]. There is still no consensus regarding the best feeding practice from a bottle in order to avoid RWG and more research regarding this topic is needed.

Another possible explanation for the association between bottle-feeding and RWG found may be the content of the bottle and the relatively high intake of protein associated with formula milk [19]. Newer formula milk products with a lower and better protein quality have been developed [20], but they still contain a higher protein quantity than does breast milk.

Nighttime meals containing formula milk were positively associated with RWG during the first 0–3-4 and 0–6 months while breastmilk given at night were negatively associated with RWG. Besides risk factors associated with bottle-feeding, earlier research has shown that nighttime feeding was associated with shorter sleep duration and increased energy intake, and both factors may lead to overweight later in life [44, 45]. It has been suggested that nighttime milk may be beneficial in breastfed children but should cease after weaning [44, 46]. If nighttime feeding with formula and the associated RWG seen in our study is associated with later overweight as well and if breastmilk given at night may protect against this development need further investigation.

We also found that breastfeeding at 3–4 months was negatively associated with RWG between 0 and 3-4 months and breastfeeding at both 3–4 and 6 months were negatively associated with RWG between 0 and 6 months. This is in line with other studies from high-income countries that have shown that breastfed children have a slower weight gain trajectory than formula-fed children [17, 19]. Breastfeeding has as mentioned above, in contrast to bottle-feeding been suggested to support responsive feeding [40]. Whether the slower weight gain seen in breastfed children protects against later adiposity is debated, there are studies both in favor of [3, 47], and against that hypothesis [3, 48]. The absence of effect on adiposity which was found in the latter one, a study from Belarus have been suggested as being dependent on the reduced number of bacterial infections to which breastfeeding contributed, suggesting that the protective role of breastfeeding may be more relevant in Westernized settings [49].

Regarding the biological, socioeconomic and parental health-related risk factors for RWG this study showed that maternal weight before pregnancy, maternal gestational weight gain and paternal weight were positively associated with RWG during the first 0–3-4 months in a model adjusted for birth weight and sex. Maternal weight before pregnancy and paternal weight were risk factors for RWG during the period 0–6 months as well. Paternal BMI but not maternal BMI before pregnancy was associated with RWG during 0–3-4 and 0–6 months in this study. There are several studies that have shown associations between maternal overweight or maternal gestational weight gain [3, 8]. The importance of paternal factors for offspring overweight are still not clear, but studies have shown associations between paternal overweight and RWG [8]. It would be interesting to study in this cohort if the associations between maternal pre-pregnancy weight, gestational weight gain and parental overweight also are associated with overweight or obesity later in life. Also paternal smoking was associated with RWG between 6 and 12 months and can be considered as a socioeconomic factor, although other mechanisms could not be ruled out. Factors associated with low socioeconomic status have been connected to both RWG [15] and overweight or obesity in other studies as well [8], although the impact of paternal socioeconomic status as well as other paternal factors in relation to childhood overweight is still not fully understood [3].

Separate sensitivity analyses including the 902 boys were performed for all analyses included in this study and the main results remained in those analyses with some exceptions (Additional file 1).

A limitation of our study was the lack of more detailed information on different aspects of nutrition and feeding practices, such as volumes, exact nutritional content at every measurement point and exclusive breastfeeding. Another limitation was our exclusion of infants with missing weight data at 0, 3–4, 6 and 12 months or children weighed outside our decided age limits, which could represent a possible bias that could not be controlled for. This was mainly due to only include measurements close to the measurement points and wider limits for this would affect precision. Worth to mention is that the excluded children had a significantly lower birth weight and therefore may have had an impact on the proportion of children with RWG. There was a risk for selection bias, where children from families that chose to not participate may have been the most socially disadvantaged families with the highest frequency of children with overweight and obesity. We know from other studies that such families often decline to take part in these kind of studies [50]. Measurement error, misclassification and error in the transmission of data to the data files, were not expected to be systematic and were therefore not considered to lead to information bias. Regarding recall-bias, the questionnaires were completed at the CHCCs in connection with each of the nine measurement points and recall-bias should therefore not be a great problem. The major strength of our study was the large sample representing two thirds of all births during the study period in this region of Sweden. They were also followed prospectively over time, with a low loss to follow up. The study used standardized measurements and repeated questionnaires, reducing the risk of information bias. Furthermore, the main outcome variables were based on weight measurements carried out by trained child health care nurses strengthening the reliability of the results.

## Conclusions

We found that RWG was more common during the first 6 months of life than during the following 6 months. Bottle-feeding and nighttime meals containing formula milk were independent risk factors for RWG during the first 6 months, whereas breastfeeding during the same period was negatively associated with RWG. Our results suggest that preventive interventions against RWG should start early in life and that bottle-feeding as well as nighttime meals, both modifiable risk factors should be addressed. Furthermore, there is a need for more research regarding the best feeding practice from a bottle in order to avoid rapid weight gain.

## Supplementary information

**Additional file 1.** Sensitivity analysis, boys, Table 1-6. Sensitivity analysis with the 902 boys included in the original cohort.

## Data Availability

The datasets analysed during the current study are not publicly available. To secure the integrity of the children in the study and their families, we feel prohibited to publish raw data files online. The data file contains sensitive information, e.g. socioeconomic data and place of residence. Some parishes in the study area are too small to safeguard the total integrity of the families. Data is available from the corresponding author on reasonable request.

## Abbreviations

RWG: Rapid weight gain
SDS: Standard deviation scores
AGA: Appropriate for gestational age
SGA: Small for gestational age
MCD: Milk cereal drink
H^2^GS: The Halland health and growth study
CHCC: Child health care centers
GA: Gestational age
LGA: Large for gestational age
SD: Standard deviation
nRWG: Non rapid weight gain
