# Supplementary material for: Nutrition- and feeding practice-related risk factors for rapid weight gain during the first year of life: a population-based birth cohort study
Source: BMC Pediatr. 2020 Nov 5;20:507. doi: 10.1186/s12887-020-02391-4 (PMC7643358; doi:10.1186/s12887-020-02391-4)
Supplement: Supplementary file 1 — Additional file 1. Sensitivity analysis, boys, Table 1-6. Sensitivity analysis with the 902 boys included in the original cohort. [file 12887_2020_2391_MOESM1_ESM.docx]

**Table 1** Characteristics of the study population divided by rapid weight gain or non-rapid weight gain during 0−3-4, 0−6 and 6−12 months, sensitivity analysis

| **n (total)**  **=902 boys** | **RWG 0−3-4 months**  (n = 484) | | **nRWG 0**−**3-4 months**  (n = 418) | | | **RWG** **0**−**6 months**  (n = 437) | | **nRWG 0**−**6 months**  (n = 465) | | **RWG 6**−**12 months**  (n = 68) | | **nRWG 6**−**12 months**  (n =834) |  |
| --- | --- | --- | --- | --- | --- | --- | --- | --- | --- | --- | --- | --- | --- |
| **Birth weight^a^**  MBW ± SD (g)  Missing (n) | 3502 ± 458  12 | 3818 ± 483***  7 | | | 3502 ± 461  10 | | 3786 ± 487***  9 | | 3627 ± 436  1 | | 3651 ± 500^ns^  18 | | |
| **Size for gestational age**  SGA (n)  AGA (n)  LGA (n)  Missing (n) | 26  453  5  0 | 5  375  38  0 | | | 25  408  4  0 | | | 6  420  39  0 | 0  66  2 | | 31  762  41 | | |
| **Gestational age**  37^0^−37^6^ (n)  38^0^−40^6^ (n)  41^0^−43^5^ (n)  Missing (n)  **Maternal age**  <25 (n)  25−35 (n)  ≥35 (n)  Missing (n) | 26  348  110  0  75  325  79  5 | 14  297  107  0  52  296  66  4 | | | 25  296  116  0  65  303  64  5 | | | 15  349  101  0  62  318  81  4 | 6  39  23  0  10  45  12  1 | | 34  606  194  0  117  576  133  8 | | |
| **Mean weight gain^a^** |  | | |  |  | |  | |  | |  | | |
| 0−3-4 m (kg) | 3.1 ± 0.6 | | | 2.3 ± 0.5*** |  | |  | |  | |  | | |
| 0−6 m (kg) |  | | |  | 5.0 ± 0.5 | | 3.8 ± 0.5*** | |  | |  | | |
| 6−12 m (kg) |  | | |  |  | |  | | 3.4 ± 0.5 | | 2.0 ± 0.5*** | | |

^a^For mean values in birth weight and mean weight gain, the groups with RWG was compared with the groups with nRWG. ***p<0.001, ns, non-significant

RWG, rapid weight gain; nRWG, non-rapid weight gain; MBW, mean birth weight; SD, standard deviation; ns, non-significant; SGA, small for gestational age;

AGA, appropriate for gestational age; LGA, large for gestational age

|  | | | | | | | | | | | | | | | | | | | | | | | | |
| --- | --- | --- | --- | --- | --- | --- | --- | --- | --- | --- | --- | --- | --- | --- | --- | --- | --- | --- | --- | --- | --- | --- | --- | --- |
| **Table 2** Logistic regressions over risk factors for rapid weight gain during 0**–**3-4, 0**–**6 and 6**–**12 months, sensitivity analysis | | | | | | | | | | | | | | | | | | |  | | | | | |
|  | **RWG/** | |  |  |  | **RWG/** |  |  |  | **RWG/** | | |  | |  | | | | **n (total)** | | | | |  |
| **Risk factors** | **nRWG** | |  |  |  | **nRWG** |  |  |  | **nRWG** | | |  | |  | | | | **=902 boys** | | | | |  |
|  | **0–3-4 m (n)** | | **OR** | **95% CI** | **p** | **0–6 m (n)** | **OR** | **95% CI** | **p** | **6–12 m (n)** | | | **OR** | | | **95% CI** | | | | | | | **p** |  |
| **Birth weight** Per kg bw | | n/a | 0.24^∍^ | 0.17, 0.33 | **<0.001** | n/a | 0.28 | 0.21, 0.38 | **<0.001** | n/a | | 0.91 | | | | | | 0.55, 1.50 | | | | 0.701 | |  |
|  | |  |  |  |  |  |  |  |  |  | |  | | | | | |  | | | |  | |  |
| **Maternal education** | | |  |  |  |  |  |  |  |  | |  | | | | | |  | | | |  | |  |
| Upper secondary school | | 185/174 | 1 | Ref |  | 173/186 | 1 | Ref |  | 26/333 | | | | 1 | | | | Ref | | | |  | |  |
| Elementary school | | 27/16 | 1.48 | 0.74, 2.96 | 0.266 | 26/17 | 1.54 | 0.78, 3.03 | 0.213 | 2/41 | | | | 0.60 | | | | 0.14, 2.62 | | | | 0.497 | |  |
| University | | 252/207 | 1.07 | 0.80, 1.44 | 0.654 | 216/243 | 0.89 | 0.66, 1.19 | 0.422 | 37/422 | | | | 1.07 | | | | 0.63, 1.81 | | | | 0.804 | |  |
| Other | | 16/18 | 0.65 | 0.30, 1.40 | 0.266 | 18/16 | 1.02 | 0.48, 2.19 | 0.961 | 2/32 | | | | 0.78 | | | | 0.18, 3.48 | | | | 0.749 | |  |
|  | |  |  |  |  |  |  |  |  |  | | | |  | | | |  | | | |  | |  |
| **Paternal education** | | |  |  |  |  |  |  |  |  | | | |  | | | |  | | | |  | |  |
| Upper secondary school | | 246/214 | 1 | Ref |  | 219/241 | 1 | Ref |  | 36/424 | | | | 1 | | | |  | | | |  | |  |
| Elementary school | | 28/23 | 1.13 | 0.61, 2.10 | 0.700 | 26/25 | 1.20 | 0.65, 2.20 | 0.555 | 4/47 | | | | 1.00 | | | | 0.34, 2.95 | | | | 0.996 | |  |
| University | | 158/134 | 1.07 | 0.78, 1.46 | 0.673 | 147/145 | 1.15 | 0.85, 1.57 | 0.362 | 20/272 | | | | 0.88 | | | | 0.50, 1.56 | | | | 0.672 | |  |
| Other | | 18/20 | 0.81 | 0.40, 1.65 | 0.558 | 16/22 | 0.82 | 0.40, 1.65 | 0.572 | 6/32 | | | | 2.22 | | | | 0.87, 5.67 | | | | 0.097 | |  |
|  | |  |  |  |  |  |  |  |  |  | | | |  | | | |  | | | |  | |  |
| **Maternal smoking** | | |  |  |  |  |  |  |  |  | | | |  | |  | | | | | |  | |  |
| No | | 443/393 | 1 | Ref |  | 398/438 | 1 | Ref |  | 63/773 | | | | 1 | | | Ref | | | | |  | |  |
| Yes | | 35/21 | 0.94 | 0.52, 1.72 | 0.843 | 34/32 | 1.14 | 0.63, 2.06 | 0.666 | 4/63 | | | | 0.95 | | | 0.33, 2.76 | | | | | 0.929 | |  |
|  | |  |  |  |  |  |  |  |  |  | | | |  | |  | | | | | |  | |  |
| **Paternal smoking** | | |  |  |  |  |  |  |  |  | | | |  | |  | | | | | |  | |  |
| No | | 394/351 | 1 | Ref |  | 356/389 | 1 | Ref |  | 86/687 | | | | 1 | | | Ref | | | | |  | |  |
| Yes | | 55/38 | 1.14 | 0.71, 1.83 | 0.581 | 50/43 | 1.11 | 0.70, 1.76 | 0.664 | 7/58 | | | | 1.00 | | | 0.44, 2.26 | | | | | 0.996 | |  |
|  | |  |  |  |  |  |  |  |  |  | | | |  | |  | | | | | |  | |  |
| **Maternal weight bp** Per kg bw n/a | | | 1.01 | 0.99, 1.02 | 0.384 | n/a | 1.01 | 1.00, 1.02 | **0.046** | n/a | | | | 1.00 | | | 0.98, 1.02 | | | | | 0.728 | |  |
|  |  |  |  |  |  |  |  |  |  |  | | | |  | | |  | | |  | | | |  |
| **Maternal gwg** Per kg gw n/a | | | 1.05 | 1.02, 1.08 | **0.001** | n/a | 1.03 | 1.01, 1.06 | **0.019** | n/a | | | | 0.98 | | | 0.93, 1.03 | | | | | 0.442 | |  |
|  |  |  |  |  |  |  |  |  |  |  | | | |  | | |  | | | | |  | |  |
| **Paternal weight** Per kg bw | | n/a | 1.02 | 1.01, 1.04 | **0.001** | n/a | 1.02 | 1.01, 1.03 | **0.003** | n/a | | | | 1.00 | | | 0.98, 1.02 | | | | 0.894 | | |  |
|  | |  |  |  |  |  |  |  |  |  |  | | | | (Continues) | | | | | | | | |  |
|  | | |  |  |  |  |  |  |  |  |  | | | |  | | | |  | | | | |  |

| **Table 2** (continued) | |  |  |  |  |  |  |  |  | |  |  | | |  | | |  | |  |
| --- | --- | --- | --- | --- | --- | --- | --- | --- | --- | --- | --- | --- | --- | --- | --- | --- | --- | --- | --- | --- |
|  | **RWG/** | |  |  |  | **RWG/** |  |  |  | **RWG/** | | |  | |  | | | **n (total)** | | |
| **Risk factors** | **nRWG** | |  |  |  | **nRWG** |  |  |  | **nRWG** | | |  | |  | | | **=902 boys** | | |
|  | **0–3-4 m (n)** | | **OR** | **95% CI** | **p** | **0–6 m (n)** | **OR** | **95% CI** | **p** | **6–12 m (n)** | | | **OR** | | | | **95% CI** | **p** | |  |
| **Maternal BMI bp**  n/a | | | 0.99 | 0.96, 1.03 | 0.690 | n/a | 1.01 | 1.01, 1.04 | 0.567 | | n/a | 1,00 | | | | 0.94, 1.06 | | | 0.922 |  |
|  | | |  |  |  |  |  |  |  | |  |  | | | |  | | |  |  |
| **Paternal BMI** n/a | | | 1.05 | 1.00, 1.10 | 0.055 | n/a | 1.02 | 0.97, 1.07. | 0.390 | | n/a | 1.08 | | | | 0.72, 1.62 | | | 0.946 |  |
|  | | |  |  |  |  |  |  |  | |  |  | | | |  | | |  |  |
| **Maternal diabetes mellitus** | | |  |  |  |  |  |  |  | |  |  | | | |  | | |  |  |
| No | | 470/405 | 1 | Ref |  | 428/447 | 1 | Ref |  | |  | | | 1 | | Ref | | |  |  |
| Yes 0/4 | | | 0.00 | 0.00, - | 0.999 | 0/4 | 0.00 | 0.00, - | 0.999 | | 64/811 | | | 4.44 | | 0.44, 44.49 | | | 0.205 |  |
|  | | |  |  |  |  |  |  |  | | 1/3 | | |  | |  | | |  |  |
| **Paternal diabetes mellitus** | | |  |  |  |  |  |  |  | |  | | |  | |  | | |  |  |
| No 440/387 | | | 1 | Ref |  | 400/427 | 1 | Ref |  | |  | | | 1 | | Ref | | |  |  |
| Yes 5/5 | | | 1.09 | 0.27, 4.40 | 0.899 | 0/6 | 0.83 | 0.21, 3.27 | 0.790 | | 64/763 | | | 0.00 | | 0.00, - | | | 0.999 |  |
|  | | |  |  |  |  |  |  |  | | 0/10 | | |  | |  | | |  |  |
| **Maternal cardiovascular disease** | | |  |  |  |  |  |  |  | |  | | |  | |  | | |  |  |
| No | | 467/405 | 1 | Ref |  | 425/447 | 1 | Ref |  | |  | | | 1 | | Ref | | |  |  |
| Yes | | 5/4 | 1.08 | 0.27, 4.36 | 0.912 | 4/5 | 0.82 | 0.21, 3.24 | 0.776 | | 65/807 | | | 1.54 | | 0.19, 12.51 | | | 0.686 |  |
|  | | |  |  |  |  |  |  |  | | 1/8 | | |  | |  | | |  |  |
| **Paternal cardiovascular disease** | | |  |  |  |  |  |  |  | |  | | |  | |  | | |  |  |
| No | | 441/382 | 1 | Ref |  | 399/424 | 1 | Ref |  | | 63/760 | | | 1 | | Ref | | |  |  |
| Yes | | 2/6 | 0.36 | 0.07, 1.85 | 0.221 | 2/6 | 0.44 | 0.09, 2.22 | 0.316 | | 0/8 | | | 0.00 | | 0.00, - | | | 0.999 |  |

All risk factors were adjusted for birth weight,

RWG, rapid weight gain; nRWG, non-rapid weight gain; m, months; n, number of subjects; OR, odds ratios; 95% CI, 95% confidence intervals; p, p value; n/a, not applicable; Kg, kilogram;

Per kg bw, per kg body weight; Per kg gw, per kg gained weight; Maternal gwg, maternal gestational weight gain

| \| **Table 3** Logistic regressions over nutrition- and feeding practice-related risk factors for rapid weight gain during 0**–**3 months, sensitivity analysis \| \| \| \| \| \| \| \| \| \| \| \| \| \| \| \| --- \| --- \| --- \| --- \| --- \| --- \| --- \| --- \| --- \| --- \| --- \| --- \| --- \| --- \| --- \| \|  \| \| **n in** \| \| --- \|   **model** \| **Model 1** \| \| \|  \| **Model 2** \| \| \|  \| **Model 3 n (total)** \| \| \| \| \| \| **Risk factors**  **0–3 m** \| **OR** \| **95% CI** \| **p** \| **n in**  **model** \| **OR** \| **95% CI** \| **p** \| **n in**  **model** \| **OR** \| \| **95% CI** \| **=902 boys**  **p** \| \| \| **Breastfeeding** \|  \|  \|  \|  \|  \|  \|  \|  \|  \| \|  \|  \| \|  \| \| 0 months \| 874 \|  \|  \|  \| 806 \|  \|  \|  \| 685 \| \|  \|  \| \|  \| \| No \|  \| 1 \| Ref \|  \|  \| 1 \| Ref \|  \|  \| \| 1 \| Ref \| \|  \| \| Yes \|  \| 0.62 \| 0.33, 1.17 \| 0.140 \|  \| 0.54 \| 0.27, 1.10 \| 0.090 \|  \| \| 0.58 \| 0.27, 1.26 \| \| 0.167 \| \|  \|  \|  \|  \|  \|  \|  \|  \|  \|  \| \|  \|  \| \|  \| \| 3**–**4 months \| 870 \|  \|  \|  \| 801 \|  \|  \|  \| 680 \| \|  \|  \| \|  \| \| No \|  \| 1 \| Ref \|  \|  \| 1 \| Ref \|  \|  \| \| 1 \| Ref \| \|  \| \| Yes \|  \| 0.45 \| 0.31, 0.65 \| **<0.001** \|  \| 0.41 \| 0.27, 0.62 \| **<0.001** \|  \| \| 0.38 \| 0.24, 0.60 \| \| **<0.001** \| \|  \|  \|  \|  \|  \|  \|  \|  \|  \|  \| \|  \|  \| \|  \| \|  \|  \|  \|  \|  \|  \|  \|  \|  \|  \| \|  \|  \| \|  \| \| **Bottle-feeding** \|  \|  \|  \|  \|  \|  \|  \|  \|  \| \|  \|  \| \|  \| \| 0 months \| 835 \|  \|  \|  \| 771 \|  \|  \|  \| 680 \| \|  \|  \| \|  \| \| No \|  \| 1 \| Ref \|  \|  \| 1 \| Ref \|  \|  \| \| 1 \| Ref \| \|  \| \| Yes \|  \| 1.77 \| 1.26, 2.49 \| **0.001** \|  \| 1.95 \| 1.36, 2.81 \| **<0.001** \|  \| \| 2.01 \| 1.34, 3.01 \| \| **0.001** \| \|  \|  \|  \|  \|  \|  \|  \|  \|  \|  \| \|  \|  \| \|  \| \| 3**–**4 months \| 649 \|  \|  \|  \| 592 \|  \|  \|  \| 505 \| \|  \|  \| \|  \| \| No \|  \| 1 \| Ref \|  \|  \| 1 \| Ref \|  \|  \| \| 1 \| Ref \| \|  \| \| Yes \|  \| 1.34 \| 0.96, 1.89 \| 0.087 \|  \| 1.39 \| 0.96, 1.99 \| 0.078 \|  \| \| 1.37 \| 0.91, 2.05 \| \| 0.129 \| \|  \|  \|  \|  \|  \|  \|  \|  \|  \|  \| \|  \|  \| \|  \| \|  \|  \|  \|  \|  \|  \|  \|  \|  \|  \| \|  \|  \| \|  \| \| **Nighttime meals** \|  \|  \|  \|  \|  \|  \|  \|  \|  \| \|  \|  \| \|  \| \| 3**–**4 months \| 649 \|  \|  \|  \| 803 \|  \|  \|  \| 682 \| \|  \|  \| \|  \| \| No \|  \| 1 \| Ref \|  \|  \| 1 \| Ref \|  \|  \| \| 1 \| Ref \| \|  \| \| Yes \|  \| 0.79 \| 0.55, 1.16 \| 0.229 \|  \| 0.82 \| 0.54, 1.23 \| 0.328 \|  \| \| 0.80 \| 0.50, 1.23 \| \| 0.298 \|   Model 1, adjusted for birth weight; Model 2, additionally adjusted for maternal and paternal education and maternal and paternal smoking; Model 3,  additionally adjusted for maternal weight before pregnancy, maternal gestational weight gain, paternal weight at the first measurement point and  maternal or paternal diabetes mellitus and cardiovascular disease  **Table 4** Logistic regressions over nutrition- and feeding practice-related risk factors for rapid weight gain during 0**–**6 months, sensitivity analysis | | | | | | | | | | | | | | |
| --- | --- | --- | --- | --- | --- | --- | --- | --- | --- | --- | --- | --- | --- | --- | --- | --- | --- | --- | --- | --- | --- | --- | --- | --- | --- | --- | --- | --- | --- | --- | --- | --- | --- | --- | --- | --- | --- | --- | --- | --- | --- | --- | --- | --- | --- | --- | --- | --- | --- | --- | --- | --- | --- | --- | --- | --- | --- | --- | --- | --- | --- | --- | --- | --- | --- | --- | --- | --- | --- | --- | --- | --- | --- | --- | --- | --- | --- | --- | --- | --- | --- | --- | --- | --- | --- | --- | --- | --- | --- | --- | --- | --- | --- | --- | --- | --- | --- | --- | --- | --- | --- | --- | --- | --- | --- | --- | --- | --- | --- | --- | --- | --- | --- | --- | --- | --- | --- | --- | --- | --- | --- | --- | --- | --- | --- | --- | --- | --- | --- | --- | --- | --- | --- | --- | --- | --- | --- | --- | --- | --- | --- | --- | --- | --- | --- | --- | --- | --- | --- | --- | --- | --- | --- | --- | --- | --- | --- | --- | --- | --- | --- | --- | --- | --- | --- | --- | --- | --- | --- | --- | --- | --- | --- | --- | --- | --- | --- | --- | --- | --- | --- | --- | --- | --- | --- | --- | --- | --- | --- | --- | --- | --- | --- | --- | --- | --- | --- | --- | --- | --- | --- | --- | --- | --- | --- | --- | --- | --- | --- | --- | --- | --- | --- | --- | --- | --- | --- | --- | --- | --- | --- | --- | --- | --- | --- | --- | --- | --- | --- | --- | --- | --- | --- | --- | --- | --- | --- | --- | --- | --- | --- | --- | --- | --- | --- | --- | --- | --- | --- | --- | --- | --- | --- | --- | --- | --- | --- | --- | --- | --- | --- | --- | --- | --- | --- | --- | --- | --- | --- | --- | --- | --- | --- | --- | --- | --- | --- | --- | --- | --- | --- | --- | --- | --- | --- | --- | --- | --- | --- | --- | --- | --- | --- | --- | --- | --- | --- | --- | --- | --- | --- | --- | --- | --- | --- | --- | --- | --- | --- | --- | --- | --- | --- | --- | --- | --- | --- | --- | --- | --- | --- | --- | --- | --- | --- | --- | --- | --- | --- | --- | --- | --- | --- | --- | --- | --- | --- | --- | --- | --- | --- | --- | --- | --- | --- | --- | --- | --- | --- | --- | --- | --- | --- | --- | --- | --- | --- | --- | --- | --- | --- | --- | --- | --- | --- | --- | --- | --- | --- | --- | --- | --- | --- | --- | --- | --- | --- | --- | --- | --- | --- | --- | --- | --- | --- | --- | --- | --- | --- | --- | --- | --- | --- | --- | --- | --- | --- | --- | --- | --- | --- | --- | --- | --- | --- | --- | --- | --- | --- | --- | --- | --- | --- | --- | --- | --- | --- | --- | --- |
|  | \| **n in** \| \| --- \|   **model** | **Model 1** | | |  | **Model 2** | | |  | **Model 3 n (total)** | | | |  |
| **Risk factors**  **0–6 m** |  | **OR** | **95% CI** | **p** | **n in**  **model** | **OR** | **95% CI** | **p** | **n in**  **model** | **OR** | | **95% CI** | **=902 boys**  **p** |  |
| **Breastfeeding** |  |  |  |  |  |  |  |  |  | |  |  |  |  |
| 0 months | 874 |  |  |  | 806 |  |  |  | 685 | |  |  |  |  |
| No |  | 1 | Ref |  |  | 1 | Ref |  |  | | 1 | Ref |  |  |
| Yes |  | 0.98 | 0.54, 1.81 | 0.955 |  | 0.92 | 0.48, 1.78 | 0.808 |  | | 1.04 | 0.50, 2.18 | 0.909 |  |
|  |  |  |  |  |  |  |  |  |  | |  |  |  |  |
| 3**–**4 months | 870 |  |  |  | 801 |  |  |  | 680 | |  |  |  |  |
| No |  | 1 | Ref |  |  | 1 | Ref |  |  | | 1 | Ref |  |  |
| Yes |  | 0.57 | 0.40, 0.81 | **0.002** |  | 0.57 | 0.39, 0.83 | **0.004** |  | | 0.54 | 0.35, 0.83 | **0.005** |  |
|  |  |  |  |  |  |  |  |  |  | |  |  |  |  |
| 6 months | 837 |  |  |  | 767 |  |  |  | 654 | |  |  |  |  |
| No |  | 1 | Ref |  |  | 1 | Ref |  |  | | 1 | Ref |  |  |
| Yes |  | 0.58 | 0.43, 0.78 | **<0.001** |  | 0.58 | 0.42, 0.79 | **0.001** |  | | 0.59 | 0.41, 0.84 | **0.003** |  |
|  |  |  |  |  |  |  |  |  |  | |  |  |  |  |
| **Bottle-feeding** |  |  |  |  |  |  |  |  |  | |  |  |  |  |
| 0 months | 835 |  |  |  | 771 |  |  |  | 660 | |  |  |  |  |
| No |  | 1 | Ref |  |  | 1 | Ref |  |  | | 1 | Ref |  |  |
| Yes |  | 1.98 | 1.42, 2.75 | **<0.001** |  | 2.06 | 1.45, 2.94 | **<0.001** |  | | 1.64 | 1.71, 2.97 | **<0.001** |  |
|  |  |  |  |  |  |  |  |  |  | |  |  |  |  |
| 3**–**4 months | 649 |  |  |  | 592 |  |  |  | 505 | |  |  |  |  |
| No |  | 1 | Ref |  |  | 1 | Ref |  |  | | 1 | Ref |  |  |
| Yes |  | 1.81 | 1.29, 2.55 | **0.001** |  | 1.73 | 1.20, 2.48 | **0.003** |  | | 1.64 | 1.10, 2.45 | **0.016** |  |
|  |  |  |  |  |  |  |  |  |  | |  |  |  |  |
| 6 months | 869 |  |  |  | 799 |  |  |  | 680 | |  |  |  |  |
| No |  | 1 | Ref |  |  | 1 | Ref |  |  | | 1 | Ref |  |  |
| Yes |  | 1.93 | 1.38, 2.70 | **<0.001** |  | 1.90 | 1.33, 2.71 | **<0.001** |  | | 1.77 | 1.19, 2.62 | **0.005** |  |
|  |  |  |  |  |  |  |  |  |  | |  |  |  |  |
| **Nighttime meals** |  |  |  |  |  |  |  |  |  | |  |  |  |  |
| 3**–**4 months | 873 |  |  |  | 803 |  |  |  | 682 | |  |  |  |  |
| No |  | 1 | Ref |  |  | 1 | Ref |  |  | | 1 | Ref |  |  |
| Yes |  | 0.84 | 0.58, 1.22 | 0.363 |  | 0.87 | 0.59, 1.30 | 0.505 |  | | 0.88 | 0.57, 1.36 | 0.554 |  |
|  |  |  |  |  |  |  |  |  |  | |  |  |  |  |
| 6 months | 870 |  |  |  | 800 |  |  |  | 680 | |  |  |  |  |
| No |  | 1 | Ref |  |  | 1 | Ref |  |  | | 1 | Ref |  |  |
| Yes |  | 0.81 | 0.60, 1.09 | 0.161 |  | 0.78 | 0.57, 1.06 | 0.114 |  | | 0.80 | 0.56, 1.13 | 0.198 |  |

Model 1, adjusted for birth weight; Model 2, additionally adjusted for maternal and paternal education and maternal and paternal smoking; Model 3,

additionally adjusted for maternal weight before pregnancy, maternal gestational weight gain, paternal weight at the first measurement point and

maternal or paternal diabetes mellitus and cardiovascular disease

MCD, milk cereal drink, RWG, rapid weight gain; nRWG, non-rapid weight gain; m, months; n, number of subjects; OR, odds ratios; 95% CI, 95% confidence interval; p, p value

**Table 5** Logistic regressions over nutrition-and feeding practice-related risk factors for rapid weight gain during 6**–**12 months, sensitivity analysis

| **Risk factors**  **6–12 m** | **n in** | **Model 1** | | | **n in** | **Model 2** | | | **n in** | **Model 3 n (total)**  **= 902 boys** | | |
| --- | --- | --- | --- | --- | --- | --- | --- | --- | --- | --- | --- | --- |
|  | **model** | **OR** | **95% CI** | **p** | **model** | **OR** | **95% CI** | **p** | **model** | **OR** | **95% CI** | **p** |
| **Breastfeeding**  0 months |  |  |  |  |  |  |  |  |  |  |  |  |
|  | 874 |  |  |  | 806 |  |  |  | 685 |  |  |  |
| No |  | 1 | Ref |  |  | 1 | Ref |  |  | 1 | Ref |  |
| Yes |  | 0.92 | 0.32, 2.65 | 0.878 |  | 0.80 | 0.27, 2.38 | 0.685 |  | 0.66 | 0.19, 2.32 | 0.517 |
|  |  |  |  |  |  |  |  |  |  |  |  |  |
| 3-4 months | 870 |  |  |  | 801 |  |  |  | 680 |  |  |  |
| No |  | 1 | Ref |  |  | 1 | Ref |  |  | 1 | Ref |  |
| Yes |  | 1.26 | 0.64, 2.46 | 0.502 |  | 1.12 | 0.56, 2.23 | 0.758 |  | 1.72 | 0.70, 4.24 | 0.237 |
|  |  |  |  |  |  |  |  |  |  |  |  |  |
| 6 months | 837 |  |  |  | 767 |  |  |  | 654 |  |  |  |
| No |  | 1 | Ref |  |  | 1 | Ref |  |  | 1 | Ref |  |
| Yes |  | 1.46 | 0.84, 2.48 | 0.183 |  | 1.44 | 0.81, 2.56 | 0.216 |  | 1.63 | 0.82, 3.21 | 0.162 |
|  |  |  |  |  |  |  |  |  |  |  |  |  |
| MCD |  |  |  |  |  |  |  |  |  |  |  |  |
| 6 months | 823 |  |  |  | 761 |  |  |  | 646 |  |  |  |
| No |  | 1 | Ref |  |  | 1 | Ref |  |  | 1 | Ref |  |
| Yes |  | 1.00 | 0.59, 1.68 | 0.999 |  | 1.10 | 0.64, 1.89 | 0.723 |  | 0.89 | 0.47, 1.68 | 0.719 |
|  |  |  |  |  |  |  |  |  |  |  |  |  |
| **Bottle-feeding** |  |  |  |  |  |  |  |  |  |  |  |  |
| 0 months | 835 |  |  |  | 771 |  |  |  | 660 |  |  |  |
| No |  | 1 | Ref |  |  | 1 | Ref |  |  | 1 | Ref |  |
| Yes |  | 0.67 | 0.36, 1.26 | 0.218 |  | 0.63 | 0.32, 1.21 | 0.164 |  | 0.49 | 0.22, 1.12 | 0.089 |
|  |  |  |  |  |  |  |  |  |  |  |  |  |
| 3**–**4 months | 649 |  |  |  | 592 |  |  |  | 505 |  |  |  |
| No |  | 1 | Ref |  |  | 1 | Ref |  |  | 1 | Ref |  |
| Yes |  | 1.35 | 0.65, 2.78 | 0.419 |  | 1.43 | 0.68, 3.03 | 0.349 |  | 1.34 | 0.57, 3.14 | 0.501 |
|  |  |  |  |  |  |  |  |  |  |  |  |  |
| 6 months | 869 |  |  |  | 799 |  |  |  | 680 |  |  |  |
| No |  | 1 | Ref |  |  | 1 | Ref |  |  | 1 | Ref |  |
| Yes |  | 0.99 | 0.54, 1.80 | 0.960 |  | 1.03 | 0.55, 1.92 | 0.926 |  | 0.93 | 0.47, 1.86 | 0.844 |
|  |  |  |  |  |  |  |  |  |  |  |  |  |
| **Nighttime meals** |  |  |  |  |  |  |  |  |  |  |  |  |
| 3**–**4 months | 873 |  |  |  | 803 |  |  |  | 682 |  |  |  |
| No |  | 1 | Ref |  |  | 1 | Ref |  |  | 1 | Ref |  |
| Yes |  | 0.82 | 0.44, 1.55 | 0.544 |  | 0.76 | 0.39, 1.48 | 0.413 |  | 1.16 | 0.49, 2.72 | 0.735 |
|  |  |  |  |  |  |  |  |  |  |  | (Continues) | |

**Table 5** (Continued)

| **Risk factors**  **6–12 m** | **n in** | **Model 1** | | | **n in** | **Model 2** | | | **n in** | **Model 3 n (total)**  **= 902 boys** | | |
| --- | --- | --- | --- | --- | --- | --- | --- | --- | --- | --- | --- | --- |
|  | **model** | **OR** | **95% CI** | **p** | **model** | **OR** | **95% CI** | **p** | **model** | **OR** | **95% CI** | **p** |
| **Nighttime meals** |  |  |  |  |  |  |  |  |  |  |  |  |
| 6 months | 870 |  |  |  | 800 |  |  |  | 680 |  |  |  |
| No |  | 1 | Ref |  |  | 1 | Ref |  |  | 1 | Ref |  |
| Yes |  | 0.47 | 0.28, 0.79 | **0.004** |  | 0.49 | 0.29, 0.83 | **0.008** |  | 0.54 | 0.30, 1.00 | **0.049** |

Model 1, adjusted for birth weight; Model 2, additionally adjusted for maternal and paternal education and maternal and paternal smoking;

Model 3, additionally adjusted for maternal weight before pregnancy, maternal weight gain during pregnancy, paternal weight at the first measurement point and

maternal and paternal diabetes mellitus and cardiovascular disease

MCD, milk cereal drink, RWG, rapid weight gain; nRWG, non-rapid weight gain; m, months; n, number of subjects; OR, odds ratio; 95% CI, 95%

confidence interval; p, p value

| **Table 6** Logistic regressions over content of nighttime meals and rapid weight gain during 0–3-4, 0–6 and 6–12 months, sensitivity analysis | | | | | | | | | | | | | | |
| --- | --- | --- | --- | --- | --- | --- | --- | --- | --- | --- | --- | --- | --- | --- |
| **Risk factors** | **n in**  **model** | **Model 1** | | | | **n in** | | **Model 2** | | | **n in** | **Model 3 n (total)**  **= 902 boys** | | |
|  |  | **OR** | **95% CI** | **p** | | **model** | | **OR** | **95% CI** | **p** | **model** | **OR** | **95% CI** | **p** |
| **Time period 0–3–4 months** | | |  |  | |  |  | |  |  |  |  |  |  |
|  | |  |  |  | |  |  | |  |  |  |  |  |  |
| **Nighttime meals 3–4 months** | |  |  |  | |  |  | |  |  |  |  |  |  |
| Breast milk | 873 |  |  |  | | 803 |  | |  |  | 682 |  |  |  |
| No |  | 1 | Ref | |  |  | 1 | | Ref |  |  | 1 | Ref |  |
| Yes |  | 0.53 | 0.39, 0.72 | | **<0.001** |  | 0.63 | | 0.46, 0.87 | **0.005** |  | 0.47 | 0.33, 0.69 | **<0.001** |
| Formula milk | 873 |  |  | |  | 803 |  | |  |  | 682 |  |  |  |
| No |  | 1 | Ref | |  |  | 1 | | Ref |  |  | 1 | Ref |  |
| Yes |  | 2.17 | 1.46, 3.21 | | **<0.001** |  | 2.31 | | 1.52, 3.51 | **<0.001** |  | 2.42 | 1.51, 3.87 | **<0.001** |
| Water | 873 |  |  | |  | 803 |  | |  |  | 682 |  |  |  |
| No |  | 1 | Ref | |  |  | 1 | | Ref |  |  | 1 | Ref |  |
| Yes |  | 0.98 | 0.05, 20.61 | | 0.990 |  | 0.98 | | 0.05, 20.52 | 0.989 |  | 0.96 | 0.04, 20.63 | 0.977 |
| **Time period 0–6 months**  **Nighttime meals 3–4 months** | |  |  |  | |  |  | |  |  |  |  |  |  |
| Breast milk | 873 |  |  |  | | 803 |  | |  |  | 682 |  |  |  |
| No |  | 1 | Ref | |  |  | 1 | | Ref |  |  | 1 | Ref |  |
| Yes |  | 0.63 | 0.47, 0.86 | | **0.003** |  | 0.63 | | 0.46, 0.87 | **0.005** |  | 0.63 | 0.44, 0.91 | **0.012** |
| Formula milk | 873 |  |  | |  | 803 |  | |  |  | 682 |  |  |  |
| No |  | 1 | Ref | |  |  | 1 | | Ref |  |  | 1 | Ref |  |
| Yes |  | 1.99 | 1.37, 2.91 | | **<0.001** |  | 1.99 | | 1.34, 2.97 | **0.001** |  | 2.10 | 1.34, 3.29 | **0.001** |
| Water | 873 |  |  | |  | 803 |  | |  |  | 682 |  |  |  |
| No |  | 1 | Ref | |  |  | 1 | | Ref |  |  | 1 | Ref |  |
| Yes |  | 1.21 | 0.06, 23.91 | | 0.902 |  | 1.17 | | 0.07, 20.53 | 0.916 |  | 1.44 | 0.08, 25.33 | 0.804 |
| **Nighttime meals 6 months** | |  |  | |  |  |  | |  |  |  |  |  |  |
| Breast milk | 870 |  |  |  | | 800 | |  |  |  | 680 |  |  |  |
| No |  | 1 | Ref |  | |  | | 1 | Ref |  |  | 1 | Ref |  |
| Yes |  | 0.65 | 0.49, 0.86 | **0.003** | |  | 0.64 | | 0.47, 0.86 | **0.003** |  | 0.63 | 0.45, 0.87 | **0.005** |
| MCD | 870 |  |  |  | | 800 | |  |  |  | 680 |  |  |  |
| No |  | 1 | Ref |  | |  | | 1 | Ref |  |  | 1 | Ref |  |
| Yes |  | 1.48 | 1.00, 2.20 | 0.051 | |  | 1.40 | | 0.91, 2.15 | 0.127 |  | 1.38 | 0.87, 2.21 | 0.175 |
| Water | 870 |  |  |  | | 800 | |  |  |  | 680 |  |  |  |
| No |  | 1 | Ref |  | |  | 1 | | Ref |  |  | 1 | Ref |  |
| Yes |  | 2.72 | 0.98, 7.60 | 0.056 | |  | 1.89 | | 0.66, 5.45 | 0.239 |  | 1.90 | 0.65, 5.61 | 0.243 |
|  |  |  |  |  | |  |  | |  |  |  |  | (Continues) | |
|  | |  |  |  | |  |  | |  |  |  |  |  |  |
| **Table 6** (Continued) | |  |  |  | |  |  | |  |  |  |  |  |  |
|  |  |  |  |  | |  |  | |  |  |  |  |  | **n (total)** |
| **Risk factors** | **n in** |  | **Model 1** |  | | **n in** | |  | **Model 2** |  | **n in** |  | **Model 3** | **=902 boys** |
|  | **model** | **OR** | **95% CI** | **p** | | **model** | | **OR** | **95% CI** | **p** | **model** | **OR** | **95% CI** | **p** |
| **Time period 6–12 months** | |  |  |  | |  |  | |  |  |  |  |  |  |
| **Nighttime meals 3-4 months** | |  |  |  | |  |  | |  |  |  |  |  |  |
| Breast milk |  |  |  |  | |  |  | |  |  |  |  |  |  |
| No | 873 | 1 | Ref |  | | 803 | 1 | | Ref |  | 682 | 1 | Ref |  |
| Yes |  | 1.13 | 0.65, 1.97 | 0.675 | |  | 1.00 | | 0.56, 1.79 | 0.989 |  | 1.45 | 0.71, 2.97 | 0.308 |
| Formula milk | 873 |  |  |  | | 803 |  | |  |  | 682 |  |  |  |
| No |  | 1 | Ref |  | |  | 1 | | Ref |  |  | 1 | Ref |  |
| Yes |  | 0.76 | 0.37, 1.58 | 0.467 | |  | 0.87 | | 0.41, 1.82 | 0.701 |  | 0.69 | 0.28, 1.72 | 0.426 |
| Water | 873 |  |  |  | | 803 |  | |  |  | 682 |  |  |  |
| No |  | 1 | Ref |  | |  | 1 | | Ref |  |  | 1 | Ref |  |
| Yes |  | 0.00 | 0.00, - | | 0.999 |  | 0.00 | | 0.00, - | 0.999 |  | 0.00 | 0.00, - | 0.999 |

| **Nighttime meals 6 months** | |  |  |  |  |  |  |  |  |  |  |  |  |
| --- | --- | --- | --- | --- | --- | --- | --- | --- | --- | --- | --- | --- | --- |
| Breast milk | 870 |  |  |  | 800 |  |  |  | 680 |  |  |  |  |
| No |  | 1 | Ref |  |  | 1 | Ref |  |  | 1 | Ref |  |  |
| Yes |  | 0.56 | 0.33, 0.95 | **0.032** |  | 0.57 | 0.33, 0.98 | **0.041** |  | 0.67 | 0.36, 1.24 | 0.201 | |
| MCD | 870 |  |  |  | 800 |  |  |  | 680 |  |  |  | |
| No |  | 1 | Ref |  |  | 1 | Ref |  |  | 1 | Ref |  | |
| Yes |  | 0.94 | 0.45, 1.96 | 0.875 |  | 1.01 | 0.46, 2.21 | 0.983 |  | 0.93 | 0.38, 2.28 | 0.865 | |
| Water | 870 |  |  |  | 800 |  |  |  | 680 |  |  |  | |
| No |  | 1 | Ref |  |  | 1 | Ref |  |  | 1 | Ref |  | |
| Yes |  | 1.71 | 0.09, 5.44 | 0.744 |  | 0.00 | 0.00, - | 0.998 |  | 0.00 | 0.00, - | 0.998 | |

Model 1, adjusted for birth weight; Model 2, additionally adjusted for maternal and paternal education and maternal and paternal smoking; Model 3,

additionally adjusted for maternal weight before pregnancy, maternal weight gain during pregnancy, paternal weight at the first measurement point and maternal

and paternal diabetes mellitus and cardiovascular disease

MCD, milk cereal drink, RWG, rapid weight gain; nRWG; non-rapid weight gain; m, months; n, number of subjects; OR, odds ratios; 95% CI, 95% confidence

interval; p, p value
